# The Relationship Between Physician Self-Disclosure and Patient Acquisition in Digital Health Markets: Cross-Sectional Study

**DOI:** 10.2196/84963

**Published:** 2026-01-29

**Authors:** Quanchen Liu, Pengqing Yin, Jing Fan

**Affiliations:** 1 International Business School Beijing Foreign Studies University Beijing China

**Keywords:** online health communities, physician online profile, physician self-disclosure, patient decision-making, physician-patient interaction, digital health care level

## Abstract

**Background:**

Online health communities have evolved into digital marketplaces where physicians have to compete for patients. Existing research examines physician-patient dynamics through a patient-centric lens, treating physicians as passive recipients of ratings and reviews, while the strategic role of physician self-disclosure remains unexamined. This gap constrains a comprehensive understanding of how physicians can actively shape patient decisions, making the investigation of strategic self-disclosure imperative.

**Objective:**

This study aims to investigate the relationship between physician self-disclosure breadth (scope of information) and depth (detailed expertise) and patient decision-making, as well as whether regional digital health care level (DHL) moderates these relationships.

**Methods:**

We conducted a cross-sectional analysis of observational data to test these relationships. Data were collected from China’s online health care platform Haodf from September to December 2024. Self-disclosure breadth (including clinical performance, academic experience, and social reputation), self-disclosure depth (including expertise coverage, richness, and granularity), and patient decision-making (total visits) were captured through manual content coding and quantitative measurement. We used structured content analysis to extract the disclosure components, informational scope, and descriptive details of each profile. Then, using validated operational formulas, we calculated the composite indices for disclosure breadth and depth based on the coded dimensions. The study generated 1798 final physician samples with complete data across 14 focal variables. The hypotheses were tested using an ordinary least squares regression model, and 4 robustness checks were conducted, including variable substitution and different resampling techniques.

**Results:**

In the primary ordinary least squares regression models, self-disclosure breadth was significantly and positively associated with patient visits (β=0.255, 95% CI 0.054-0.456; *P=*.01), as was self-disclosure depth (β=0.098, 95% CI 0.030-0.167; *P*=.005). The breadth×DHL interaction was positive and significant (β=0.261, 95% CI 0.061-0.461; *P*=.01). Similarly, the depth×DHL interaction was positive and significant (β=0.070, 95% CI 0.002-0.138; *P*=.045). It should be noted that the association for self-disclosure breadth was stronger than that of self-disclosure depth. DHL strengthened the relationship between the disclosure strategies with patient visits. This contextual amplification indicates that DHL serves as a critical boundary condition, determining the degree to which physician self-disclosure strategies translate into patient acquisition outcomes.

**Conclusions:**

This study reconceptualizes physicians as strategic agents shaping patient decision-making through purposeful self-disclosure. Different from existing studies treating physicians as passive recipients of ratings and reviews, our research demonstrates that physicians can strategically shape patient acquisition through self-disclosure breadth and depth. This study brings new insights to digital health markets by demonstrating that self-disclosure operates as a viable patient acquisition mechanism, wherein the DHL acts as a critical boundary condition. The findings have real-world implications: (1) physicians can leverage evidence-based disclosure strategies, (2) platforms should implement context-adaptive features, and (3) policymakers should prioritize digital infrastructure investments to enhance physicians' competitive capabilities and patient decision-making quality.

## Introduction

### Background

The health care landscape is experiencing an unprecedented digital transformation, with online health communities (OHCs) emerging as powerful intermediaries that fundamentally reshape patient-physician interactions. OHCs democratize medical information access and empower patients to actively evaluate health care providers before making consultation decisions [1]. Platforms, such as HealthTap [2] and China’s Haodf, now serve millions of users globally, with the OHCs market projected to expand from US $13.3 billion in 2022 to US $42.9 billion by 2030 [3]. This revolution forces physicians to build compelling online presences beyond clinical excellence to compete effectively in an increasingly crowded digital marketplace.

### Review of Relevant Scholarship

Existing research on OHCs has predominantly examined physician-patient dynamics through a patient-centric lens, treating physicians as passive recipients of online ratings and reviews rather than strategic actors capable of influencing patient decisions. Current studies focus extensively on how patients leverage physician profiles, ratings, and accumulated reviews to screen health care providers [4], effectively positioning physicians as static entities whose past digital footprints predetermine patient selection outcomes. This perspective fundamentally overlooks physicians’ potential for active agency in patient acquisition. Moreover, while scholars acknowledge regional variations in digital health care level (DHL [5,6]), encompassing the sophistication of technological infrastructure, digital literacy capabilities, and information accessibility within specific health care environments), little attention has been paid to how these contextual differences might alter physicians’ strategic opportunities and patients’ information processing capabilities, creating a significant theoretical blind spot in understanding physician behavior within digitally heterogeneous health care environments.

In an increasingly crowded OHC landscape where patients can choose from hundreds of providers, physicians must now strategically differentiate themselves through deliberate self-presentation beyond clinical excellence and accumulated reviews. Within this context, self-disclosure theory from social psychology offers a promising framework, suggesting that strategic information disclosure across breadth (scope of information) and depth (level of detail) dimensions can enhance credibility, build trust, and reduce decision-making uncertainty [7], thereby influencing differential patient choices. However, the effectiveness of physician self-disclosure cannot be understood in isolation from regional digital health care contexts. In technologically advanced regions, physicians access sophisticated multimedia tools that enhance disclosure opportunities, while patients possess higher digital literacy, enabling effective interpretation of complex professional information [8]. Conversely, less digitally developed areas present technological constraints limiting physicians’ ability to communicate expertise effectively, while patients may lack sufficient digital literacy to process and verify disclosed information [9]. This contextual complexity suggests that physician self-disclosure effectiveness may vary across digital health care environments, as strategies proving highly effective in advanced contexts might yield diminished returns in regions with limited infrastructure and lower digital literacy.

### Aims, Objectives, and Hypotheses

#### Overview

Based on these research gaps and theoretical considerations, our research aims to contribute to the existing literature on physician strategic self-disclosure behavior in OHCs by addressing 2 primary research objectives. First, we aim to investigate how physician self-disclosure breadth and depth are associated with patient decision-making in OHCs. Second, we seek to determine whether and how the regional DHL moderates the relationship between these self-disclosure strategies and patient choices.

#### Physician Self-Disclosure and Patient Decision-Making

Literature review in Section 1 in Multimedia Appendix 1 highlights that physicians’ self-disclosure significantly shapes patients’ credibility and trust toward decision-making by providing multifaceted professional information. Within OHCs, both breadth and depth dimensions of physician self-disclosure systematically activate these cognitive evaluations, ultimately shaping patient consultation decisions.

Self-disclosure breadth enhances patient decision-making by providing comprehensive professional signals that directly build credibility. When physicians disclose extensive information across multiple professional dimensions, they create a rich tapestry of verifiable cues that patients can cross-reference and validate. This comprehensive presentation first enhances perceived source credibility, as patients can observe concrete evidence of qualifications across diverse professional domains, reducing concerns about physician competence. The breadth of disclosure subsequently fosters interpersonal trust by signaling transparency and professional openness, suggesting that physicians have “nothing to hide” and are confident in their professional standing. Finally, this extensive information scope significantly increases perceived diagnostic value by providing patients with sufficient data points to make informed assessments about physician-patient compatibility. Patients can evaluate whether the physician’s experience, training, and achievements align with their specific medical needs and preferences, thereby reducing decision-making uncertainty and increasing consultation likelihood.

Self-disclosure depth is associated with patient decision-making through intensive information quality that demonstrates specialized expertise and professional communication. When physicians provide detailed expertise descriptions, they signal profound clinical knowledge and commitment to patient understanding. This depth reinforces perceived source credibility by showcasing mastery within specific medical domains, as detailed explanations indicate genuine expertise rather than superficial knowledge. It also then builds interpersonal trust by demonstrating physicians’ investment in clear communication and patient education, suggesting benevolent intentions and professional dedication. Most critically, depth maximizes perceived diagnostic value by enabling patients to precisely evaluate treatment fit—detailed specialty descriptions allow patients to determine whether their specific conditions fall within the physician’s demonstrated areas of expertise. This granular matching capability reduces ambiguity about treatment appropriateness and increases patients’ confidence in scheduling consultations with physicians.

#### DHL as the Moderator

DHL systematically shapes the mechanisms through which self-disclosure breadth and depth operate, fundamentally altering how the same self-disclosure content is produced, transmitted, and interpreted.

Advanced DHL amplifies the credibility-building effects of self-disclosure breadth through enhanced verification mechanisms and seamless information processing. When DHL is high, physicians can populate comprehensive profile fields with verifiable credentials, embed direct links to official registries, and present information through user-friendly interfaces that facilitate patient navigation. Patients in these contexts possess the digital literacy to efficiently cross-validate credentials through integrated databases and verification systems, creating a low-friction pathway for establishing source credibility. This enhanced verification capability strengthens the breadth-credibility relationship posited by self-disclosure theory, thereby accelerating patients' cognitive progression from enhanced source credibility to ultimate consultation decisions. In contrast, regions with limited digital infrastructure constrain verification processes, weakening the credibility signals that breadth disclosure would otherwise provide.

Similarly, advanced DHL intensifies the trust-building effects of self-disclosure depth by enabling rich multimedia presentations and sophisticated patient interpretation capabilities. High-level digital health care environments allow physicians to create comprehensive and well-structured disclosure experiences. Patients with elevated digital literacy can effectively parse these complex multimedia presentations, interpreting granular clinical details and structured expertise descriptions as authentic signals of both professional competence and patient-centered communication. This enhanced processing capability amplifies the depth-trust relationship, as patients can fully appreciate the nuanced expertise demonstrations that deep disclosure provides. Conversely, in regions with poor connectivity and limited digital literacy, deep disclosure content may fail to render properly or overwhelm patients’ interpretive capacities, potentially undermining rather than enhancing the intended trust-building effects. On the basis of the preceding discussion, we advance the following two hypotheses:

- Hypothesis 1: Physician self-disclosure breadth and depth are positively associated with patient decision-making.
- Hypothesis 2: DHL positively moderates the relationship between self-disclosure depth, breadth, and patient decision-making.

## Methods

### Sample Size, Power, and Precision

We conducted a cross-sectional study that was designed and reported in accordance with the JARS (Journal Article Reporting Standards) guidelines [10] to examine the relationship between physician self-disclosure and patient acquisition in digital health markets. Our cross-sectional secondary analysis aimed to estimate the association between physicians’ online self-disclosure and patient acquisitions with an absolute precision of ±0.5 percentage points at a 95% CI. The required number of physician profiles was calculated with the single-proportion formula sample size (n)=[(*Z*_(1-_*_α_*_/2)_)^2^*P*(1-*P*)]/*d*^2^ [11-13], where *Z*_(1-_*_α_*_/2)_=the critical value with a corresponding standard level of confidence (1.96 at 95% CI), *P* was the conservative prevalence of 50% (as this value maximizes variance when the true prevalence is unknown and therefore yields the largest required sample size, ensuring adequate power and precision [14]), d=5% allowable margin of error or desired precision, indicating a minimum sample size of 384 physicians. To guard against unforeseen data-quality issues, we set a conservative target of at least 1000 evaluable records. Besides, this study is observational and contains no experimental aims, power calculations for between-group comparisons were unnecessary.

### Data Collection

Haodf (established in 2006) is China’s largest online health care platform, covering more than 10,000 hospitals and 900,000 physicians nationwide as of July 2023. Physician participation is exceptionally high, with 280,000 physicians registered under verified real names to deliver online consultation services, rendering the platform a unique and well-suited context for investigating physician–patient interactions [15].

Guided by the platform’s interface, we deployed a crawler to systematically extract information from the public profiles of 2050 physicians from September to December 2024. After rigorous data cleaning and outlier removal, we followed the coding protocol established by Herzenstein et al [16] to quantify physicians’ self-disclosure. Four trained research assistants independently coded fourteen dichotomous disclosure variables. The dataset was split into 2 equal batches, with each assigned to a distinct pair of coders who worked in parallel. Only observations with unanimous agreement were retained, and all cases of coder disagreement were excluded. This intercoder validation yielded 1798 reliable observations for subsequent empirical analysis, with no missing values in the final analytic dataset—comfortably exceeding our preregistered target of 1000 evaluable records.

### Variable Measurements

#### Dependent Variable

We used “Total visits”—the cumulative number of consultations, calls, and bookings shown on each physician’s profile—as the dependent variable. This variable, therefore, provides a comprehensive foundation for examining the relationship between physician self-disclosure and patient decision-making. All variable measurement details can be found in Table S1 in Section 2 of Multimedia Appendix 1.

#### Independent Variable

Self-disclosure breadth was indexed by entropic weighting of 3 public signals, namely clinical performance (cp), academic experience (ae), and social reputation (sr). This composite measure captures the comprehensiveness of physicians’ self-presentation strategies by integrating these fundamental aspects of credible identity. Self-disclosure depth was also indexed by entropic weighting of 3 important cues, such as expertise coverage (ec), expertise richness (er), and expertise granularity (eg). These 3 dimensions collectively capture the multifaceted nature of disclosure depth, as physicians may vary in how extensively they elaborate (coverage), how comprehensively they describe (richness), and how specifically they detail their expertise (granularity). Specifically, self-disclosure breadth and depth were calculated using the following formulas:

Self-disclosure breadth = log (α _1_ * cp + α _2_ * ae + α_3_ * sr + 1)

Self-disclosure depth = log (α_4_ * ec + α_5_ * er + α_6_ * eg + 1)

#### Moderating Variable

DHL measures the extent of digital technology integration within a city’s health care infrastructure, reflecting the adoption of telemedicine platforms and mobile clinical tools in routine medical practice [17]. We operationalized this variable using the China Urban Digital Economy Index (Medical Chapter) [18], which is the most up-to-date DHL-relevant index dataset we can find, as a comprehensive assessment jointly published by the School of Management at Zhejiang University and the Digital Economy Research Centre of New H3C Group. Based on this index, each city’s DHL is assigned a score from 1 to 5, with higher numbers representing higher levels of digital integration and technological advancement in digital health care. We assigned each physician the DHL score corresponding to their practice location, thereby capturing the digital maturity of their local digital health care environment, as detailed in Table S2 in Section 2 of Multimedia Appendix 1.

#### Controls

To separate the associations of physician self-disclosure from other factors related to patient selection, we included three control variables—professional title (title), physician popularity (popularity), and gift (gift)—to account for alternative explanations of patient decision-making. A brief summary of variable measurements is presented in Table 1.

**Table 1:** Brief summary of measurement of core study variables.

Variables		Measurements
**Dependent variable**		
	Total visits	The cumulative count of all patient-initiated interactions with each physician across all service channels (online consultations, telephone consultations, and appointment bookings), as recorded on the platform.
**Independent variable**		
	Self-disclosure breadth	Coded as 1 if at least one clinical component (clinical experience, clinical effectiveness, and clinical manner) was disclosed, and 0 otherwise.
	Clinical performance	Coded as 1 if at least one academic component (research productivity, international training, and educational credentials) was disclosed, and 0 otherwise.
	Academic experience	Coded as 1 if at least one social component (part-time positions, honors and awards) was disclosed, and 0 otherwise.
**Self-disclosure depth**		
	Expertise coverage	The total length of description text in vocabularies.
	Expertise richness	The number of disease types explicitly mentioned as areas of expertise in the physician’s profile.
	Expertise granularity	Coded as 1 if the description uses delimiters to distinguish specialties, and 0 otherwise.
**Moderator**		
	Digital health care level	Each physician is assigned a score from 1 to 5 corresponding to their practice location.
**Controls**		
	Title	Chief physicians receive 4, deputy chief physicians 3, attending physicians 2, and resident physicians 1.
	Popularity	A composite recommendation score (0-5 continuous scale) generated by the platform.
	Gift	Patients’ real payment to the physician after receiving services. Take the records on the platform.

### Modeling and Statistical Analysis

#### Research Model

**Figure 1:**
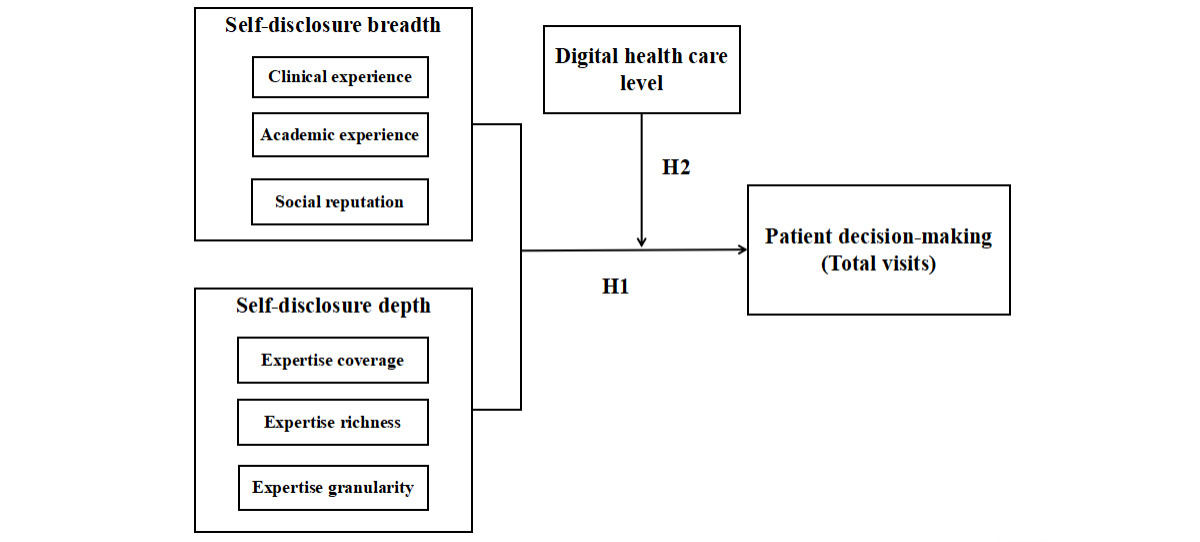
Research model.

Figure 1 depicts our research model. This study examines the relationship between physicians’ self-disclosure breadth and depth and patient decision-making in digital health markets (H1) and the moderating role of DHL in these relationships (H2).

#### Ordinary Least Squares Model

Before testing the main and moderating effects, we first performed a descriptive statistical analysis to summarize the key characteristics of physicians and their self-disclosure behaviors. Subsequently, we conducted correlation analysis to assess the associations between key variables of interest. We then tested the main effects, focusing on how self-disclosure breadth and depth shape patient decision-making. Following this, we evaluated the moderating role of DHL, investigating whether regional digital health care conditions shape the effectiveness of these self-disclosure strategies. The empirical models pertaining to these tests were as follows:

- Model 1: Ln(Total visits) = β0 + β1Self-disclosure breadth + β2Title + β3Popularity + β4 Gift + ε
- Model 2: Ln(Total visits) = β0 + β1Self-disclosure depth + β2Title + β3Popularity + β4Gift + ε
- Model 3: Ln(Total visits) = β0 + β1Self-disclosure breadth + β2DHL + β3Self-disclosure breadth× DHL + β4Title + β5Popularity + β6Gift + ε
- Model 4: Ln(Total visits) = β0 + β1Self-disclosure depth + β2DHL + β3Self-disclosure depth × DHL + β4 Title + β5 Popularity + β6 Gift + ε

Our models include 3 control variables, including physician title, popularity metrics, and gift reception status, with *ε* representing the error term. We control for title because prior studies indicate that professional credentials significantly affect patient trust and provider selection [19]. Popularity serves as an important platform-based signal that may independently drive patient choices [20]. Gift reception reflects patient satisfaction from previous interactions, potentially influencing patients’ future decision-making patterns [21].

We used ordinary least squares regression to estimate all models, with data analysis conducted in Stata (StataCorp LLC). To ensure comparability across variables with different scales, all variables were standardized before regression analysis.

### Ethical Considerations

The Institutional Review Board of the International Business School, Beijing Foreign Studies University (001-2025-12-02) approved this study and granted an exemption from full human-subjects review. This study was conducted in accordance with the national ethical guidelines for research involving information data. Our use of legally obtained, fully anonymized public data, which contained no sensitive or commercial elements, qualified for an exemption from full ethics review as stipulated by the National Health Commission of the People’s Republic of China [22]. No further approvals were required.

Researchers also confirmed that the original data-collection practices of Haodf are governed by the platform’s user agreement and privacy notice, and private fields, such as physician names, clinical records, were anonymized and deidentified from the analytic file. No additional recruitment with physicians or patients occurred, no payments or incentives were offered to any physician or patient, and no identification of individual participants in any images of the manuscript or supplementary material is possible.

## Results

### Descriptive Statistics

Table 2 summarizes the central tendencies and dispersion of the key variables drawn from 1798 physician profiles on the Haodf platform. Min-Max denotes the actual minimum and maximum values in the sample. The IQR is calculated as the difference between the 75th and 25th percentiles and is reported alongside the median (50th percentile). The results showed a skewed distribution of total visits per physician (mean 1471.83, SD 2486.38). In the subsequent data-processing pipeline, we applied appropriate normalization steps to mitigate the impact of extreme values and ensure the analytic dataset is well-behaved. Some physicians claim competence in clinical experience (mean 0.46, SD 0.50) and effectiveness (mean 0.32, SD 0.47), but only 4% (72/1798) explicitly mention clinical manner (mean 0.04, SD 0.20).

**Table 2:** Descriptive statistics of focal variables.

Variable			Mean (SD)		Median		Range
Total visits			1471.83 (2486.38)		680.5		1-27,352
**Self-disclosure depth**							
	Expertise richness	3.76 (1.66)		4		0-8	
	Expertise coverage	8.16 (5.72)		7		0-58	
	Expertise granularity	0.51 (0.50)		1		0-1	
**Self-disclosure breadth**							
	Clinical experience	0.46 (0.50)		0		0-1	
	Clinical effectiveness	0.32 (0.47)		0		0-1	
	Clinical manner	0.04 (0.20)		0		0-1	
	Research productivity	0.75 (0.43)		1		0-1	
	International training	0.48 (0.50)		0		0-1	
	Educational credentials	0.60 (0.49)		1		0-1	
	Part-time positions	0.67 (0.47)		1		0-1	
	Honors and awards	0.44 (0.50)		0		0-1	
Digital health care level			4.09 (1.01)		4		1-5
Title			3.35 (0.72)		3		1-4
Gift			128.65 (303.06)		39		0-4878
Popularity			4.14 (0.310)		4.1		3.4-5

Research productivity is reported by 75% (1349/1798) throughout the sample (mean 0.75, SD 0.43), whereas 48% (863/1798) list international training (mean 0.48, SD 0.50) and 60% (1079/1798) cite elite educational credentials (mean 0.60, SD 0.50). Roughly two-thirds hold part-time positions (mean 0.67, SD 0.47) and 44% (791/1798) have earned honors or awards (mean 0.44, SD 0.50). The average DHL is 4.09 (SD 1.01), and the mean title rank is 3.35 (SD 0.72), both approaching the upper end of their respective scales. Finally, popularity—an index computed by the platform—clusters tightly around 4.14 (SD 0.31), implying limited variance once the algorithmic score is normalized.

### Pearson Correlation Analysis and Collinearity Testing

Table S3 in Section 2 of Multimedia Appendix 1 reports the Pearson correlations for 6 focal variables entering regression. Both self-disclosure depth and breadth are positively related to total visits (*r*=0.098; *P*<.001 and *r*=0.109; *P*<.001, respectively). The moderator DHL also positively relates to the dependent variable (*r*=0.119; *P*<.001). The VIF (variance inflation factor) analysis shows, afterwards in Table S4 in Section 2 of Multimedia Appendix 1, the largest value is 2.82 for gift, followed by 1.67 for popularity, whereas the remaining VIFs range only from 1.06 to 1.11. All VIF values are far below the conventional threshold of 5 (or 10) [23]. Taken together, the correlation matrix and the inflation factors jointly indicate that multicollinearity should not be a main concern for the stability or estimation of regression results.

### Hypothesis Testing

We estimated 4 ordinary least squares models with heteroskedasticity-robust standard errors. The dependent variable, Total visits, was log-transformed to reduce skewness. All core continuous predictors, including self-disclosure breadth, self-disclosure depth, and DHL, were standardized (mean 0, SD 1) to facilitate coefficient comparability and to avert multicollinearity when interaction terms were introduced. Across all 4 models, the coefficients show the relative change in total visits associated with a one-standard-deviation shift in the focal variable. The results are reported in Table 3.

**Table 3:** Ordinary least squares regression results (Models 1-4) examining the relationship between physician self-disclosure breadth, depth, digital health care level, and patient decision-making.

Variable	Model 1	Model 2	Model 3	Model 4
Self-disclosure breadth, β (95% CI)	0.255^a^ (0.054-0.456)	0.249^a^ (0.047-0.450)	—^b^	—
Self-disclosure depth, β (95% CI)	—	—	0.098^c^ (0.030-0.167)	0.092^c^ (0.023-0.160)
DHL^d^, β (95% CI)	—	–0.036 (–0.09 to 0.018)	—	–0.031 (–0.085 to 0.023)
Self-disclosure breadth×DHL, β (95% CI)	—	0.261^a^ (0.061-0.461)	—	—
Self-disclosure depth×DHL, β (95% CI)	—	—	—	0.070^a^ (0.002-0.138)
Title, β (95% CI)	0.248^c^ (0.172-0.324)	0.242^c^ (0.166-0.317)	0.276^c^ (0.201-0.350)	0.275^c^ (0.201-0.349)
Popularity, β (95% CI)	1.720^c^ (1.50-1.94)	1.751^c^ (1.532-1.970)	1.678^c^ (1.460-1.900)	1.700^c^ (1.480-1.921)
Gift, β (95% CI)	0.001^c^ (0.001-0.001)	0.001^c^ (0.001-0.001)	0.001^c^ (0.001-0.001)	0.001^c^ (0.001-001)
Constant, β (95% CI)	–1.825^c^ (–2.720 to –0.930)	–1.938^c^ (–2.843 to –1.031)	–1.748^c^ (–2.648 to –0.847)	–1.838^c^ (–2.749 to –0.926)
*F* test (*df*)	251.3 (6)	181.4 (8)	251.9 (6)	181.1 (8)
*R* ^*2*^	0.412	0.415	0.413	0.415

^a^The correlation is significant at a significance level of .05 (2-tailed).

^b^Not applicable.

^c^The correlation is significant at a significance level of .01 (2-tailed).

^d^DHL: digital health care level.

Model 1 establishes the baseline relationship for self-disclosure breadth, revealing a positive and statistically significant coefficient (β=0.255, 95% CI 0.054-0.456; *P*=.01), thereby confirming Hypothesis 1 that broader physician self-disclosure increases patient decision-making volume. Model 3 demonstrates a parallel finding for self-disclosure depth, with results showing a significant positive effect (β=0.098, 95% CI 0.030-0.167; *P*=.005), providing support for Hypothesis 1 that physician self-disclosure depth is positively associated with patient decision-making. Both findings confirm that comprehensive information disclosure, whether through diverse disclosure topics or detailed expertise presentation, enhances physician attractiveness to patients.

The interaction analyses reveal that DHL significantly amplifies self-disclosure effectiveness. Model 2 introduces the breadth×DHL interaction term, yielding a positive and significant coefficient (β=0.261, 95% CI 0.061-0.461; *P*=.01), which supports Hypothesis 2 that DHL strengthens the relationship between self-disclosure breadth and patient decision-making. Similarly, Model 4 demonstrates that the depth×DHL interaction is positive and significant (β=0.070, 95% CI 0.002-0.138; *P*=.045), corroborating Hypothesis 2 that DHL enhances the effectiveness of disclosure depth. Notably, the breadth interaction effect is substantially larger than the depth interaction effect, suggesting that DHL provides greater amplification benefits for diverse disclosure strategies compared to detailed expertise presentation.

### Post Hoc Analysis

**Figure 2:**
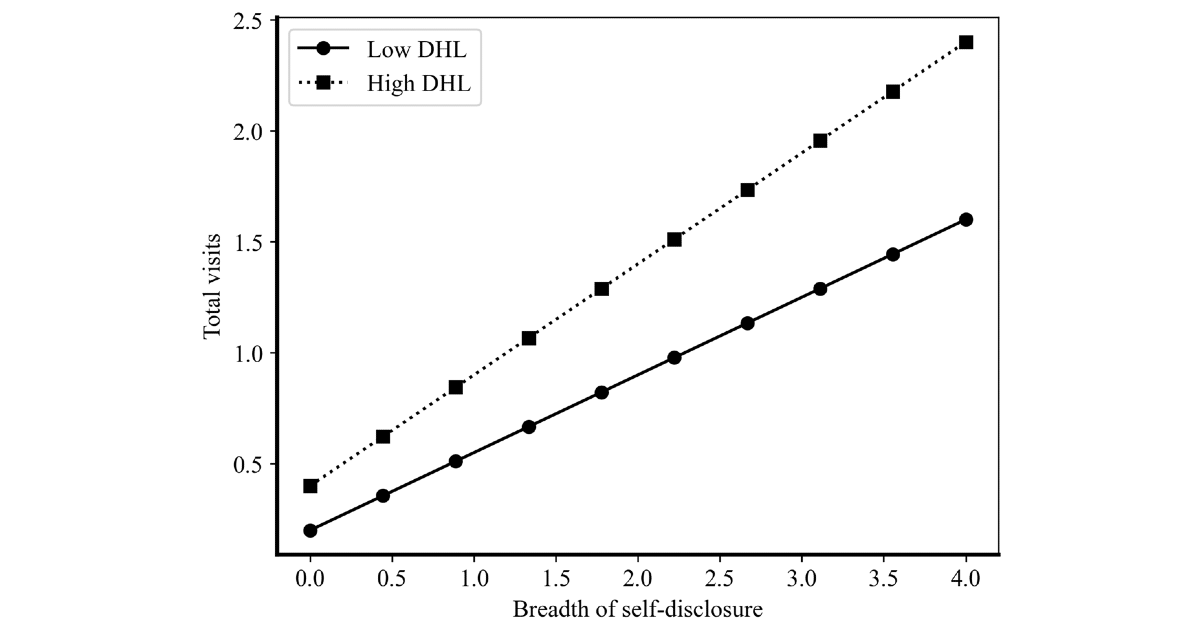
The moderating effect of digital health care level on the association between physician self-disclosure breadth and patient decision-making. DHL: digital health care level.

**Figure 3:**
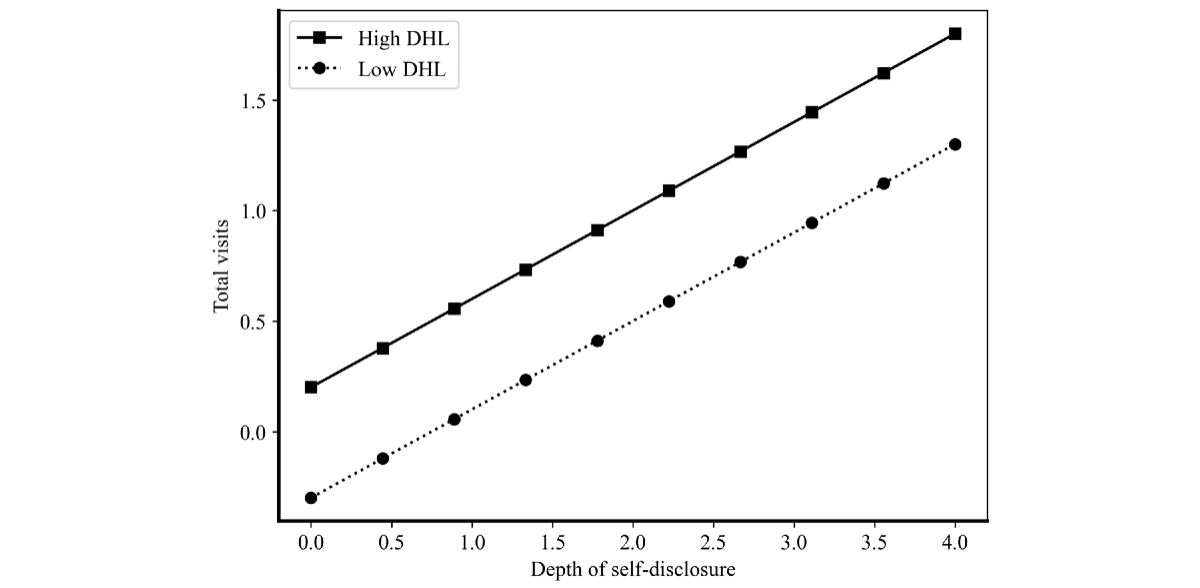
The moderating effect of digital health care level on the association between physician self-disclosure depth and patient decision-making. DHL: digital hhealth care level.

To better understand the nuanced mechanisms underlying the moderating effects of DHL, we conducted post hoc analysis using interaction plots. While our main regression results demonstrate statistically significant moderation effects, visualizing these interactions provides deeper insights into how DHL moderates the strength and nature of the relationships between physician self-disclosure strategies and patient decision-making. The visualized graphs are presented in Figures 2 and 3, where high and low DHL correspond to values of 1 SD above and below the mean (+1 and –1).

Figure 2 reveals particularly prominent insights about breadth moderation. Most notably, the gap between high and low DHL conditions grows increasingly larger as self-disclosure breadth increases, creating a pronounced divergence pattern that suggests breadth disclosure may be more sensitive to DHL than depth disclosure. In high DHL environments, patients appear exceptionally responsive to broad professional presentations. This amplified responsiveness may reflect enhanced information processing capabilities and greater appreciation for diverse professional information. Conversely, in low DHL environments, increasing breadth yields relatively restricted returns, potentially due to limited digital literacy or infrastructure constraints that prevent effective usage of comprehensive professional profiles. The steeper moderation gap for breadth compared to depth suggests that while diverse disclosure topics (breadth) become disproportionately valuable when supported by higher DHL, detailed expertise presentations (depth) maintain more consistent effectiveness across different DHL contexts. This creates a “digital divide” effect where technological advancement in health care becomes a critical prerequisite for breadth disclosure effectiveness.

Figure 3 provides compelling visual evidence for the DHL moderation effect on self-disclosure depth, revealing a striking divergence in self-disclosure effectiveness across DHL levels. In high DHL environments, the relationship between self-disclosure depth and total visits exhibits a pronounced higher slope, demonstrating that each incremental increase in depth disclosure generates greater patient engagement gains. Conversely, in low DHL contexts, the relationship remains relatively lower, suggesting that detailed expertise presentations yield fewer additional benefits when digital health care is underdeveloped. This pattern indicates that physicians practicing in technologically advanced health care environments can leverage detailed self-disclosure strategies—spanning specialized medical capabilities, in-depth professional competencies, and granular clinical expertise—to achieve disproportionately greater patient attraction, while those in less digitized environments may find such detailed disclosure strategies less rewarding.

### Robustness Checks

We used various methods for robustness checks to ensure the reliability and consistency of our research findings, including variable substitution, bootstrap resampling, subsampling, and the winsorizing technique. The core findings are summarized in Table 4 below. The procedures and interpretations for each approach are detailed below.

To ensure the reliability of our findings, we first conducted a robustness check by replacing our categorical DHL measurement with continuous digital health care scores, which were also presented in the report published by China Urban Digital Economy Index (Medical Chapter) [18], as shown in Table S5 of Section 3 in Multimedia Appendix 1. The detailed results of this first variable substitution check are shown immediately afterwards in Table S6, demonstrating consistent patterns across all model specifications. Self-disclosure breadth continues to be positively associated with total visits across Models 1 and 2 (β=0.255, 95% CI 0.054-0.456; *P*=.01 and β=0.258, 95% CI 0.057-0.459; *P*=.01, respectively), while self-disclosure depth demonstrates similar consistency in Models 3 and 4 (β=0.098, 95% CI 0.030-0.167; *P*=.005 and β=0.096, 95% CI 0.027-0.164; *P*=.006, respectively). The preservation of both effect magnitudes and significance levels indicates that our core findings are not artifacts of the initial categorical operationalization.

To rule out the possibility that physicians attract more consultations simply because they are more active or popular, we further re-estimated all models by replacing the dependent variable with Ln (total visits-per-popularity, ie, total visits divided by popularity), where Popularity is a platform-computed index that combines physician activity, patient recommendations, and review ratings to reflect overall physician popularity. Table S7 shows that the core disclosure variables remain positively significant across the 4 specifications, with Models 1 and 2 (β=0.494, 95% CI 0.290-0.699; *P*<.001 and β=0.482, 95% CI 0.277-0.687; *P*<.001, respectively); and Models 3 and 4 (β=0.157, 95% CI 0.086-0.228; *P*<.001 and β=0.151, 95% CI 0.080-0.222; *P*<.001, respectively). The persistent significance of self-disclosure after controlling for popularity corroborates our main results and demonstrates that, even after adjusting for physicians’ baseline visibility and popularity, patients show a preference for physicians who provide greater breadth and depth of professional information.

To validate that our significance tests are not dependent on distributional assumptions, we re-estimated all models using bootstrap resampling with 1000 replications. Table S8 presents the bootstrapped results, demonstrating the robustness of our statistical inferences. First, self-disclosure breadth maintains its positive and significant relationship with total visits in both Models 1 and 2 (β=0.255, 95% CI 0.050-0.460; *P*=.02 and β=0.249, 95% CI 0.049-0.448; *P*=.02, respectively), while self-disclosure depth demonstrates identical significance patterns in Models 3 and 4 (β=0.098, 95% CI 0.294-0.167; *P*=.005 and β=0.092, 95% CI 0.026-0.258; *P*=.006, respectively). Besides, the moderation effects remain significant under bootstrap estimation. The breadth×DHL interaction retains its positive and significant coefficient (β=0.261, 95% CI 0.054-0.467; *P*=.01) in Model 2, while the depth×DHL interaction similarly maintains significance (β=0.070, 95% CI 0.007-0.133; *P*=.03) in Model 4.

To address potential concerns that our main findings might be driven by senior physicians who possess inherently greater credibility and resources, we conduct a robustness check by restricting our analysis to non–chief physicians only. As shown in Table S9, the detailed results of subsample analysis (non–chief physicians) show consistent effects across the restricted sample, confirming the robustness of our main findings. Self-disclosure breadth maintains its positive and significant relationship with total visits in both Models 1 and 2 (β=0.345, 95% CI 0.074-0.615; *P*=.01 and β=0.345, 95% CI 0.075-0.614; *P*=.01 respectively), while self-disclosure depth similarly shows robust positive effects in Models 3 and 4 (β=0.125, 95% CI 0.032-0.219; *P*=.009 and β=0.124, 95% CI 0.029-0.215; *P*=.01, respectively). Notably, the coefficient magnitudes are actually larger in this subsample compared to the full sample, suggesting that self-disclosure strategies may be even more crucial for physicians with lower hierarchies.

To address potential concerns about extreme values influencing our results, we re-estimated all models after winsorizing the top and bottom 10% of each variable. Table S10 presents the detailed results from this outlier-treatment approach. Self-disclosure breadth maintains its positive and significant effects across Models 1 and 2 (β=0.250, 95% CI 0.030-0.470; *P*=.03 and β=0.248, 95% CI 0.028-0.468; *P*=.03, respectively), while self-disclosure depth similarly preserves its significant positive relationship in Models 3 and 4 (β=0.106, 95% CI 0.032-0.180; *P*=.005 and β=0.101, 95% CI 0.027-0.175; *P*=.008, respectively). The coefficient magnitudes remain virtually identical to our original estimates, confirming that extreme values do not drive the main effect conclusions.

**Table 4:** A brief summary of the focal results of our 5 robustness tests.

Robust check		Self-disclosure breadth, β (95% CI)	Self-disclosure depth, β (95% CI)	Self-disclosure breadth×DHL^a^, β (95% CI)	Self-disclosure depth×DHL, β (95% CI)
**R1^b^**					
	Model 1	0.255^c^ (0.054-0.456)	__^d^	__	__
	Model 2	0.258^c^ (0.057-0.459)	__	0.020^e^ (0.006-0.034)	__
	Model 3	__^c^	0.098^e^ (0.030-0.167)	__	__
	Model 4	__	0.096^e^ (0.027-0.164)	__	0.006^c^ (0.001-0.010)
**R2^f^**					
	Model 1	0.494^e^ (0.290-0.699)	__	__	__
	Model 2	0.482^e^ (0.277-0.687)	__	0.030^g^ (0.002-0.063)	__
	Model 3	__	0.157^e^ (0.086-0.228)	__	__
	Model 4	__	0.151^e^ (0.080-0.222)	__	0.071^g^ (0.001-0.142)
**R3^h^**					
	Model 1	0.255^c^ (0.050-0.460)	__	__	__
	Model 2	0.249^c^ (0.049-0.448)	__	0.261^c^ (0.054-0.467)	__
	Model 3	__	0.098^e^ (0.029-0.167)	__	__
	Model 4	__	0.092^e^ (0.026-0.258)	__	0.070^c^ (0.007-0.133)
**R4^i^**					
	Model 1	0.345^c^ (0.074-0.615)	__	__	__
	Model 2	0.345^c^ (0.075-0.614)	__	0.259^c^ (0.001-0.518)	__
	Model 3	__	0.125^e^ (0.032-0.219)	__	__
	Model 4	__	0.124^c^ (0.029-0.215)	__	0.086^c^ (0.001-0.173)
**R5^j^**					
	Model 1	0.250^c^ (0.030-0.470)	__	__	__
	Model 2	0.248^c^ (0.028-0.468)	__	0.285^c^ (0.066-0.504)	__
	Model 3	__	0.106^e^ (0.032-0.180)	__	__
	Model 4	__	0.101^e^ (0.027-0.175)	__	0.076^c^ (0.003-0.149)

^a^DHL: digital health care level.

^b^Replace categorical DHL measurement with continuous digital health care scores.

^c^The correlation is significant at a significance level of .05 (2-tailed).

^d^Not applicable.

^e^The correlation is significant at a significance level of .01 (2-tailed).

^f^Replace the dependent variable with Log_e_ (total visits-per-popularity).

^g^The correlation is significant at a significance level of .1 (2-tailed).

^h^Re-estimated all models using bootstrap resampling with 1000 replications.

^i^Restricted our analysis to non–chief physicians only.

^j^Re-estimated all models after winsorizing the top and bottom 10% of each variable.

## Discussion

### Principal Findings

This study demonstrates that physicians can strategically shape patient decision-making through purposeful self-disclosure behaviors within OHCs. Our empirical analysis from China’s leading online health platform reveals that both self-disclosure breadth and depth significantly increase patient visits, challenging the prevailing view of physicians as passive recipients of online reviews. Most critically, the regional DHL fundamentally moderates these relationships. In cities with advanced digital development, both breadth and depth effects are substantially amplified, while regions with limited digital development show diminished returns for the same disclosure strategies. These findings reconceptualize physician agency within digital health care platforms, demonstrating that strategic self-disclosure represents a viable patient acquisition mechanism rather than passive information provision.

### Theoretical Implications

The study advances theoretical understanding of physician self-disclosure in digital health markets by embedding its 3 key contributions within—and explicitly contrasting them against—the extant literatures on physician agency, self-disclosure theory, and OHCs.

First, contrary to prior studies that predominantly frame physicians as passive recipients of online feedback [4,24,25], our reconceptualization of physician agency aligns with a growing but still underdeveloped stream of research that emphasizes the proactive role of service providers in digital platforms. For instance, recent work by Ouyang and Wang [26] and Lu and Wu [27] suggests that physicians can influence patient perceptions through profile customization and online engagement. This challenges the prevailing passive-physician paradigm in medical marketing research and establishes physicians as active agents capable of patient acquisition through evidence-based self-presentation strategies [28,29]. However, extant research has predominantly conceptualized online reputation management as a reactive endeavor—centering on prompt responses to negative feedback [30], remediation of service failures [31], or post hoc optimization of profile completeness [32]—while overlooking proactive self-disclosure breadth and depth as ex ante instruments that physicians can strategically deploy to attract new patients before any review is written. Our findings go beyond these accounts by revealing that physicians can deploy proactive, ex ante self-disclosure—systematically foregrounding personal credentials, institutional affiliations, and succinct expertise narratives—to pre-emptively sculpt patient trust and choice before any review is written or any service failure occurs. This reorients the theoretical lens from “damage control” to “impression engineering,” recasting self-disclosure as a forward-looking signaling mechanism that anticipates patient heuristics rather than remedying prior dissatisfaction. By establishing strategic disclosure as a feasible alternative to reactive reputation management, we push the conceptual boundary of physician behavior in digital environments beyond traditional service-recovery frameworks and toward a predictive, marketing-as-signal paradigm.

Second, we extend self-disclosure theory beyond its traditional interpersonal communication context into health care settings while establishing critical boundary conditions [7,33,34]. We introduce the regional DHL as a critical boundary condition—a factor largely overlooked in prior self-disclosure research. While earlier studies have examined individual-level moderators, such as gender [35] or cultural orientation [36], our findings reveal that macrolevel technological development significantly moderates the disclosure-outcome relationship. This aligns with recent macrosociological perspectives on digital inequality [37], which argue that the same online behavior can yield divergent outcomes depending on the technological context in which it is embedded. Thus, our study not only extends self-disclosure theory into a new domain but also redefines its boundary conditions by incorporating sociotechnological contingencies. Our findings confirm that self-disclosure breadth and depth operate in professional contexts, yet their effectiveness is contingent on the regional DHL. By demonstrating this macrolevel technological moderation, we extend the theory’s scope and expose context boundaries previously overlooked [38,39].

Third, the prevailing view in the OHC literature maintains that these platforms mainly mitigate physician-patient information asymmetry by aggregating ratings, reviews, and outcome data [24,40,41]. We extend this perspective by demonstrating that physicians can actively reconfigure the information environment through strategic self-disclosure. Especially, our findings show that, within Chinese OHCs, breadth of disclosure markedly outweighs depth, as an expansive array of credential signals consistently exhibits a greater positive association with patient engagement than rich narrative detail does. Patients appear to follow a hierarchical signaling model in which credential heuristics operate as an initial, low-cognitive gatekeeper, with hospital tier, academic rank, and prestigious awards are rapidly recoded into a binary “pass-fail” filter that determines inclusion in the consideration set. Only after clearing this threshold do patients allocate scarce attentional resources to elaborately process narrative depth—articulations of treatment philosophy, detailed case histories, or other discursive evidence of clinical expertise. Empirically, the stronger main effect of self-disclosure breadth compared to depth evidences that, in the context of Chinese OHCs, patients may initially rely more heavily on credential heuristics—such as titles, affiliations, and awards [42,43]—as efficient signals of quality, before engaging with the more cognitively demanding narratives of detailed expertise. This insight complements and extends recent work by Wang et al [44], who find that physicians with more comprehensive profiles receive more appointment requests, thereby offering a mechanistic account of why breadth outperforms depth in Chinese OHCs.

### Practical Implications

This study also provides several practical implications for multiple stakeholder groups seeking to enhance physician self-disclosure effectiveness and patient decision-making within OHCs.

For physicians, our findings emphasize a context-dependent approach to online self-disclosure [45-47]. Those in high-DHL regions should leverage comprehensive breadth—showcasing credentials across clinical, academic, and social domains—and enrich their profiles with multimedia depth, such as video introductions. In low-DHL regions, the priority shifts to maximizing clarity. Physicians should focus on core breadth elements like clinical experience and use concise, text-based depth with scannable lists of expertise to ensure accessibility.

For OHC platform designers, our results highlight the limitation of a uniform profile design and advocate for context-aware systems [48,49]. Actionable recommendations include implementing structured disclosure templates to guide physicians in highlighting decision-critical information, introducing visual verification badges for credentials to enhance trust, and developing tiered interface modes—a feature-rich version for high-DHL users and a streamlined, text-optimized version for regions with limited DHL.

For policymakers, our evidence on the moderating role of DHL underscores that digital infrastructure is a social determinant of health access. Specific interventions should prioritize closing the digital divide by investing in high-speed internet infrastructure in underserved areas, launching public health campaigns to improve patients’ digital health literacy, and creating financial incentives for clinics and physicians in low-DHL regions to adopt and master digital consultation tools.

### Limitations

This study acknowledges certain limitations. First, the cross-sectional design limits causal inference, leaving the temporal dynamics of these relationships unclear. Second, the DHL measurement uses a city-level categorization rather than granular technological indicators, which prevents identifying the specific infrastructure components that most critically moderate disclosure effectiveness. Third, owing to the absence of more recent publicly available datasets, the DHL indicators used in this study remain those published in 2021. The 3-year lag may introduce slight discrepancies with present-day infrastructure levels. Updated figures can further corroborate our findings. Finally, focus on China’s online health platform may limit generalizability across different cultural and regulatory contexts. The findings may reflect sociocultural norms specific to Chinese health care markets, such as a pronounced hierarchy in physician-patient dynamics or a strong preference for credential-based trust signals.

### Conclusions

This study is innovative in reconceptualizing physicians as strategic agents capable of actively shaping patient decision-making through purposeful self-disclosure in digital health markets. Different from existing studies that treat physicians as passive recipients of online ratings and reviews, our research demonstrates that physicians can strategically shape patient acquisition through deliberate self-disclosure breadth and depth. This study thus brings new insights to digital health markets by demonstrating that self-disclosure operates as a viable patient acquisition mechanism in professional health care contexts, wherein DHL acts as a critical boundary condition that fundamentally moderates the breadth-depth relationships. The findings have significant implications in the real world: (1) physicians can leverage evidence-based disclosure strategies for patient acquisition, (2) platform designers should implement context-adaptive features optimizing effectiveness across heterogeneous digital environments, and (3) policymakers should prioritize digital infrastructure investments to systematically enhance physicians’ competitive capabilities and patient decision-making quality.

## Appendix

Multimedia Appendix 1 Details on literature review, variable measurements and robustness check results.

## Abbreviations

DHL: digital health care level
JARS: Journal Article Reporting Standards
OHC: online health community
VIF: variance inflation factor

## Data Availability

The datasets generated during and/or analyzed during this study are available from the corresponding author on reasonable request.
