# Supplementary material for: The Relationship Between Physician Self-Disclosure and Patient Acquisition in Digital Health Markets: Cross-Sectional Study
Source: J Med Internet Res. 2026 Jan 29;28:e84963. doi: 10.2196/84963 (PMC12902760; doi:10.2196/84963)
Supplement: Multimedia Appendix 1 [file jmir_v28i1e84963_app1.docx]

## Multimedia Appendix

## Section 1: Literature Review

### Online health communities

Amidst rapid technological advancements and pervasive digital transformation across industries, Online Health Communities (OHCs) have emerged and flourished as influential platforms in the healthcare landscape [1]. Confronted with complex medical decisions — ranging from selecting hospitals to determining appropriate treatment plans — patients gain access to a convenient, information-rich platform through OHCs [2]. These platforms enable them to explore a wealth of information, including other patients’ treatment experiences and professional evaluations of physicians, thereby allowing patients to make decisions that align more closely with patients’ individual needs and preferences [3].

Traditional healthcare settings are characterized by pronounced information asymmetry between physicians and patients [4]. This asymmetry stems from physicians’ specialized knowledge and clinical expertise, which places them in an informationally advantaged position while relegating patients to a passive role. OHCs help address this fundamental imbalance by providing transparent, comprehensive physician profiles [5]. These profiles encompass multiple dimensions of professional information, serving as two critical functions. First, these profiles serve as a public showcase for physicians to actively demonstrate their clinical capabilities and professional competence [6]. Second, it facilitates patients to identify physicians whose expertise specifically aligns with their medical conditions [7], thereby improving both treatment relevance and potential effectiveness. Consequently, this transparency enhances patient trust while reducing the uncertainty that typically results from information gaps in traditional healthcare encounters.

Given these transparency benefits of OHCs, existing research has predominantly examined physician-patient dynamics through a patient-centric lens, treating physicians as passive recipients of online visibility rather than active agents in patient acquisition. A substantial body of work has explored how patients leverage OHCs to screen physicians before initial consultations, showing that online ratings, textual reviews, and physician-generated content effectively help patients identify providers whose expertise and interpersonal approaches align with their specific needs [8]. This patient-focused perspective is evident in studies that typically measure how accumulated rating scores or review sentiment subsequently influence appointment bookings [9] and shape patients’ trust perceptions of unfamiliar physicians [10]. Even research examining physicians’ online disclosures maintains this passive framework: when physicians voluntarily share credentials or respond to forum questions, such activities are characterized as static information provision that increases patient trust and reduces uncertainty [11].

This understanding of physician agency becomes even more complex when considering the contextual variations. Current OHCs research typically assumes that platform benefits operate uniformly across all contexts, treating comprehensive profile systems and communication channels as having a consistent impact regardless of the broader digital healthcare landscape [12]. However, the same platform features may yield varying effectiveness depending on the technological and social contexts in which they are embedded [13]. The reality suggests a more nuanced dynamic where contextual factors may create differential opportunities for strategic physician behaviour, influencing both physician capabilities for effective self-presentation and patient capacities for information processing within these digital platforms.

### Self-disclosure theory

Self-disclosure is defined as the act of selectively revealing personal information, including personal experiences, emotional states, inner thoughts, or secrets, to others during social interactions [14]. This constitutes a voluntary and active process of information sharing. Self-disclosure emerged in the 1960s as social psychologists increasingly focused on the formation and development of interpersonal relationships [15]. It elucidates the underlying psychological mechanisms through which disclosed information influences decision-making [16].

Self-disclosure theory primarily identifies two key dimensions in the self-disclosure act: breadth and depth [17]. The breadth of self-disclosure generally refers to the scope of information disclosed, including education, job title, and work experience [18]. Meanwhile, the depth of self-disclosure pertains to the privacy or core aspects of the information, particularly within one’s sensitive and intimate domain [17,19]. These dimensions have been consistently adopted and validated across various research contexts over the past five decades. For instance, physicians’ online biographies coded for breadth (range of credentials, publications, affiliations) and depth (self-reflection on clinical challenges, patient-care philosophy) have been shown to predict patient trust and appointment intentions [20]. Subsequent organizational-behaviour research extended breadth to encompass multiple résumé-like categories (certifications, awards, prior roles) and depth to sensitive disclosures about failures, ethical dilemmas, or personal motivations [21]. More recently, in online settings, scholars have measured breadth via the number of distinct profile fields completed on social networking sites (e.g., LinkedIn’s education, skills, endorsements) and depth via the inclusion of intimate narratives or vulnerability-laden stories [22].

Specifically, in the context of OHCs, the strategic self-disclosure of medical experiences and professional knowledge by physicians is identified as an important mechanism influencing patient decision-making [23]. On the one hand, the breadth of physicians’ self-disclosure can significantly enhance their perceived credibility, creating a positive cascade effect on patient choices [24]. When physicians provide extensive information across multiple professional dimensions patients develop a more complete understanding of their expertise and qualifications. For example, a physician who not only identifies their role as an internal medicine specialist at a prestigious hospital but also introduces their medical training, professional titles, and experience at renowned institutions is likely to be perceived as highly knowledgeable and professionally versatile [25]. This enhanced credibility perception directly translates into greater patient trust [26], increasing the likelihood that patients will prioritize offline services with that physician over their original healthcare plans [27].

Beyond the breadth dimension, the depth of physicians’ self-disclosure also contributes to patient decision-making. When physicians provide detailed insights into their core professional competencies — such as innovative approaches [28], specialized techniques to complex conditions, and engagement with cutting-edge medical research — they demonstrate not merely credentials but active expertise and clinical value [29]. For instance, a physician who elaborates on their successful management of rare diseases or utilization of advanced medical technologies effectively showcases mastery within their specialty, which helps bridge the knowledge gap between physician and patient while fostering deeper trust [30]. This trust, built upon demonstrated competence rather than formal qualifications alone, encourages patients to engage more openly in discussing their conditions and concerns.

While self-disclosure theory’s breadth and depth dimensions have been validated across interpersonal and organizational contexts, their application to physician-patient interactions in OHCs reveals critical theoretical gaps. Current research adopts a predominantly patient-centric perspective, treating physicians as passive information providers. This approach fundamentally misaligns with self-disclosure theory’s core premise, which positions disclosure as a strategic, voluntary process designed to cultivate specific relational outcomes. Moreover, existing studies fail to account for how regional variations in digital infrastructure, which alter physicians’ controllability over disclosure strategies.

### Digital healthcare level

The rise of digital healthcare technologies has fundamentally reshaped patient-physician interactions, introducing contextual variations that significantly impact the effectiveness of Online Health Communities (OHCs) [31]. Digital healthcare level (DHL) represents a critical construct that captures these contextual differences, encompassing the sophistication of technological infrastructure, digital literacy capabilities, and information accessibility within specific healthcare environments [32]. As healthcare systems increasingly adopt digital solutions, DHL serves as an important moderating factor that determines how effectively physicians can leverage self-disclosure strategies and how competently patients can process and utilize disclosed information within OHCs.

DHL functions through three interconnected mechanisms that systematically reshape both physicians’ disclosure capabilities and patients’ information-processing capacities. First, the sophistication of technological infrastructure directly influences physicians’ strategic disclosure opportunities by granting them access to advanced multimedia tools. These tools enable richer, more nuanced communication, thereby expanding the range and depth of information that can be effectively conveyed to patients [33,34]. In high-DHL environments, physicians can create rich, verifiable profiles with embedded links to official credentials and real-time performance metrics, while low-DHL contexts limit disclosure to basic text formats with minimal verification capabilities. Second, population digital literacy levels fundamentally shape patients’ ability to effectively interpret and validate disclosed information [35]. In digitally advanced settings, patients are equipped with the ability to verify credentials via integrated databases and interpret complex professional content, whereas those in less developed digital environments often face challenges in processing sophisticated information or confirming its authenticity [36]. Third, information accessibility infrastructure influences the speed and reliability with which disclosed information can be transmitted and accessed, creating differential user experiences that influence the self-disclosure evaluation process [37].

These differential capabilities create boundary conditions for self-disclosure theory’s application in healthcare contexts. In regions with advanced DHL, robust verification mechanisms amplify the credibility-building effects of breadth disclosure, while sophisticated multimedia capabilities enhance the trust-building potential of depth disclosure. Conversely, regions with limited DHL may experience diminished returns from strategic disclosure efforts, as technological constraints and literacy limitations impede both physician presentation capabilities and patient interpretation processes. This contextual dependency challenges the assumption of uniform self-disclosure effectiveness across healthcare environments and highlights the need to examine DHL as a critical moderating factor in physician-patient interactions within OHCs.

## Section 2: Variable Measurements

### Dependent variable

We selected “total visits” as our dependent variable to measure patient decision-making. This metric, extracted directly from each physician’s public profile, represents the cumulative count of all patient-initiated interactions, including online consultations, telephone consultations, and appointment bookings.

Total visits serve as an informative measure for our research objectives for three key reasons. First, it captures revealed patient preferences through actual selection behaviour: patients actively choose to engage with specific physicians after evaluating available information, making this metric a direct reflection of demand. Second, it provides comprehensive coverage across all service modalities offered on the platform, avoiding the truncation bias that would result from examining any single interaction type. Third, it functions as a reliable proxy for favourable word-of-mouth and service quality within the platform’s ecosystem, as sustained patient engagement typically reflects positive service experiences and outcomes.

This measure therefore provides a robust and comprehensive foundation for examining the relationship between physician self-disclosure and patient decision-making.

### Independent variable

We operationalized self-disclosure breadth through three core dimensions that comprehensively capture the information physicians disclose to influence patient selection: clinical performance, academic experience, and social reputation. This framework reflects the multifaceted nature of physician credibility as perceived by patients in online healthcare environments.

Clinical performance encompasses practical competence indicators such as years of practice, case volume, and treatment outcomes, providing patients with direct evidence of the physician’s ability to address their medical needs effectively. Academic experience includes educational credentials, research publications, and specialized training, signalling intellectual rigor and expertise in handling complex medical cases. Social reputation incorporates professional honours and external recognition, offering social validation and trust signals that help patients navigate the inherent uncertainty of healthcare decisions. These three dimensions collectively represent the primary information categories that patients systematically evaluate when selecting physicians online, providing a comprehensive yet parsimonious framework for measuring self-disclosure breadth in digital healthcare contexts.

Specifically, Clinical performance (Cp) represents physicians’ practical competence through three key indicators: clinical experience, clinical effectiveness, and clinical manner. This dimension captures the hands-on aspects of medical practice that directly relate to patient care quality and treatment outcomes. Clinical performance is operationalized as a binary variable calculated using the following formula:

$$\text{Cp=}\left\{ \begin{aligned} \text{ 0, if no clinical performance }\text{disclosure} \\ \text{1, if at least one clinical component disclosed} \end{aligned} \right.$$

This binary coding approach reflects whether physicians choose to disclose any information about their practical clinical capabilities. A value of 0 indicates complete absence of clinical performance disclosure across all three indicators, while a value of 1 signifies that the physician has provided information in at least one clinical performance domain, demonstrating their engagement in showcasing practical competence to potential patients.

Academic experience (Ae) represents physicians’ scholarly preparation and intellectual engagement through three key indicators: research productivity, international training, and educational credentials. This dimension captures the academic and educational aspects of medical expertise that demonstrate continuous learning, research involvement, and exposure to diverse medical practices. Academic experience is operationalized as a binary variable calculated using the following formula:

$$\text{Ae=}\left\{ \begin{aligned} \text{0, if no academic experience }\text{disclosure} \\ \text{1, if at least one academic component disclosed} \end{aligned} \right.$$

This binary coding approach reflects whether physicians choose to disclose any information about their academic and scholarly background. A value of 0 indicates complete absence of academic experience disclosure across all three indicators, while a value of 1 signifies that the physician has provided information in at least one academic domain, demonstrating their commitment to showcasing scholarly credentials to potential patients.

Social reputation (Sr) represents physicians’ professional standing and external recognition through two key indicators: part-time positions and honours & awards. This dimension captures the social validation and external recognition that physicians receive from the broader medical community, reflecting their established credibility and professional influence beyond direct patient care. Social reputation is operationalized as a binary variable calculated using the following formula:

$$\text{Sr=}\left\{ \begin{aligned} \text{0, if no social reputation disclosure} \\ \text{1, if }\text{at least one social component disclosed} \end{aligned} \right.$$

This binary coding approach reflects whether physicians choose to disclose any information about their professional recognition and community standing. A value of 0 indicates complete absence of social reputation disclosure across both indicators, while a value of 1 signifies that the physician has provided information in at least one reputation domain, demonstrating their effort to showcase external recognition and social validation to potential patients.

According to the above operationalization of the three core dimensions, self-disclosure breadth represents the overall diversity of professional information that physicians choose to share across clinical performance, academic experience, and social reputation. This composite measure captures the comprehensiveness of physicians’ self-presentation strategies by integrating these fundamental aspects of credible identity. Self-disclosure breadth is calculated using the following formula:

$$\text{S}\text{elf-disclosure}\text{ brea}\text{dt}\text{h }\text{=}\text{ }\text{log}\left( \text{α}_{\text{1}}\text{*}\text{ }\text{Cp}\text{ }\text{+}\text{ }\text{α}_{\text{2}}\text{*}\text{ }\text{Ae}\text{ }\text{+}\text{ }\text{α}_{\text{3}}\text{*}\text{ }\text{Sr}\text{ }\text{+}\text{ }\text{1} \right)$$

In this formula, Cp, Ae, and Sr represent Clinical performance, Academic experience, and Social reputation respectively, while *α*₁, *α*₂, and *α*₃ are weighting coefficients. To normalize the distribution and reduce the impact of outliers, we employ a logarithmic transformation. To handle cases where physicians provide no disclosure across any dimension, we add 1 within the logarithmic function to ensure mathematical validity.

On the other hand, we operationalized self-disclosure depth through three complementary dimensions that capture distinct aspects of how physicians present their expertise information on the platform. Expertise coverage (Ec) quantifies the extensiveness of information provided by measuring the total length of physicians’ descriptions of their professional expertise areas. This dimension reflects the quantitative aspect of depth—the sheer volume of text physicians dedicates to describing their capabilities, distinguishing between brief mentions and extensive elaborations. Expertise richness (Er) counts the number of specific disease types or medical conditions explicitly mentioned as areas of expertise in physicians’ online profiles. For example, a profile listing “minimally invasive treatment of lumbar disc herniation, lumbar spinal stenosis, scoliosis, spinal tuberculosis, and spinal tumours” would receive a richness score of 5. This captures the breadth-within-depth dimension, measuring how many distinct medical specializations physicians explicitly claim, reflecting the comprehensiveness of their disclosed expertise portfolio. Expertise granularity (Eg) evaluates whether physicians use structural elements such as delimiters (parentheses, semicolons, colons), cue words (“especially”, “including”, “particularly”, “e.g.”), or numbered lists to organize and distinguish their specialties. This dimension captures the organizational precision aspect of depth, measuring whether physicians present their expertise in a structured, detailed manner that facilitates patient understanding of their specific capabilities.

These three dimensions collectively capture the multifaceted nature of disclosure depth, as physicians may vary in how extensively they elaborate (coverage), how comprehensively they describe (richness), and how specifically they detail their expertise (granularity). Self-disclosure depth is calculated using the following formula:

$$\text{Self-disclosure}\text{ d}\text{ept}\text{h}\text{ }\text{=}\text{ }\text{log}\left( \text{α}_{\text{4}}\text{ }\text{*}\text{ }\text{Ec }\text{+}\text{ }\text{α}_{\text{5}}\text{ }\text{*}\text{ }\text{Er }\text{+}\text{ }\text{α}_{\text{6}}\text{ }\text{*}\text{ }\text{Eg}\text{ + }\text{1} \right)$$

In this formula, Ec, Er, and Eg represent the three depth dimensions respectively, while *α*₄, *α*₅, and *α*₆ are weighting coefficients. The formula employs the same logarithmic transformation and mathematical adjustment principles as the breadth measure.

Table S1 detailed the description and operational definition of each variable.

**Table S1.** Focal variables and their detailed operational definition (cross-sectional study of physicians on Haodf.com, China, 2024, N=1,798).

| **Name** | **Operational Definition** |
| --- | --- |
| Dependent variable | |
| Total visits | The cumulative count of all patient-initiated interactions with each physician across all service channels (online consultations, telephone consultations, and appointment bookings), as recorded on the platform. |
| **Self-disclosure breadth** | |
| Clinical performance | |
| Clinical experience | Clinical experience is manually coded as 1 if the physician’s online profile explicitly states concrete work-tenure information—such as “has practiced clinically for 3 years” — and 0 otherwise. |
| Clinical effectiveness | Clinical effectiveness is coded as 1 if the physician’s online profile contains any self-reported statement about clinical effectiveness—such as “has successfully performed more than 2,000 neurosurgical procedure” — and 0 otherwise. |
| Clinical manner | Clinical manner is coded as 1 if the physician’s online profile includes any description about clinical attitudes — such as “rigorous and conscientious” — and 0 otherwise. |
| Academic experience | |
| Research productivity | Research productivity is coded as 1 if the physician’s online profile mentions any indicators of academic output — such as research projects undertaken, papers published, books authored — and 0 otherwise. |
| International training | International training is coded as 1 if the physician’s online profile explicitly introduces any overseas exchange experience—such as “completed advanced training in surgery at the University of California, San Francisco” — and 0 otherwise. |
| Educational credentials | Educational credentials is coded as 1 if the physician’s online profile contains any mention of educational background—such as “graduated from Capital Medical University in 2013” or “earned master’s and doctoral degrees” — and 0 otherwise. |
| Social reputation | |
| Part-time positions | This variable is coded as 1 if the physician’s online profile mentions any medical-related part-time appointments—such as “Member of the Sports Medicine Section, Zhejiang Rehabilitation Medical Association”— and 0 otherwise. |
| Honors & awards | Honor & Reward is coded as 1 if the physician’s online profile lists any concrete honours or awards — such as “recipient of the Second Prize of the National Science and Technology Progress Award” — and 0 otherwise. |
| **Self-disclosure depth** | |
| Expertise coverage | Expertise coverage is measured by the total length of description text in vocabularies. |
| Expertise richness | This variable counts the number of disease types explicitly mentioned as areas of expertise in the physician’s profile. For example, if the profile lists “minimally invasive treatment of lumbar disc herniation, lumbar spinal stenosis, scoliosis, spinal tuberculosis, and spinal tumours,” the richness will be coded as 5. |
| Expertise granularity | Expertise granularity is coded as 1 if the description uses delimiters such as parentheses, semicolons, or colons, cue words such as “especially”, “including”, “particularly”, “e.g.” or numbered lists (1, (1), ①, etc.) to distinguish specialties, and 0 otherwise. |
| **Moderator** | |
| Digital healthcare level | A categorical variable (1-5 scale) measuring the extent of digital technology integration within a physician’s city, assigned according to the criteria specified in Table 1. |
| **Controls** | |
| Title | Professional title is coded on a 4-point scale where chief physicians receive a value of 4, deputy chief physicians 3, attending physicians 2, and resident physicians 1. |
| Popularity | Physician popularity is a composite recommendation score (0-5 continuous scale) algorithmically generated by the platform. |
| Gift | Patients can express their recognition and gratitude to the physician after receiving services by sending electronic gift, which requires real payment. Take the records on the platform. |

Table S2 further listed out the detailed digital healthcare level across different cities [38].

**Table S2.** Digital healthcare level across different cities (cross-sectional study of physicians on Haodf.com, China, 2024, N=1,798).

| City-level medical digitalization classification | City list | Digital healthcare Level |
| --- | --- | --- |
| Medical digitalization first-tier cities | Beijing, Shanghai, Guangzhou, Hangzhou | 5 |
| Medical digitalization new first-tier cities | Zhengzhou, Nanjing, Chengdu, Tianjin, Wuhan, Chongqing, Changsha, Xiamen, Shenzhen, Ningbo, etc | 4 |
| Medical digitalization second-tier cities | Nanchang, Nanning, Fuzhou, Suzhou, Xuzhou, Jinan, Shijiazhuang, Kunming, Ürümqi, Zhuhai, Qingdao, Wuxi, Harbin, Lanzhou, etc | 3 |
| Medical digitalization third-tier cities | Yangzhou, Luoyang, Zibo, Qinhuangdao, Taizhou, Jiujiang, Weihai, Dezhou, Chuzhou, Xingtai, Huainan, Jiangmen, Nantong, Quzhou, Linyi, Lishui, Jilin, Jiaxing, Pingdingshan, Mianyang, etc | 2 |
| Medical digitalization fourth-tier cities | Guang’an, Xuancheng, Zhanjiang, Suzhou (Anhui), Zhangjiakou, Meishan, etc | 1 |

Table S3 shows the Pearson correlations between core variables. Table S4 shows the collinearity diagnostics results.

**Table S3.** Correlations between focal study variables (cross-sectional study of physicians on Haodf.com, China, 2024, N=1,798).

| Variables | Total visits | Self-disclosure breadth | Self-disclosure depth | DHL | Title | Heat | Gift |
| --- | --- | --- | --- | --- | --- | --- | --- |
| Total visits | 1.000 | __ | __ | __ | __ | __ | __ |
| Self-disclosure breadth | 0.109^a^ | 1.000 | __ | __ | __ | __ | __ |
| Self-disclosure depth | 0.098^a^ | 0.151^a^ | 1.000 | __ | __ | __ | __ |
| DHL | 0.119^a^ | 0.090^a^ | 0.018 | 1.000 | __ | __ | __ |
| Title | 0.178^a^ | 0.228^a^ | -0.047^b^ | 0.005 | 1.000 | __ | __ |
| Heat | 0.436^a^ | 0.098^a^ | 0.155^a^ | 0.239^a^ | 0.136^a^ | 1.000 | __ |
| Gift | 0.732^a^ | 0.099^a^ | 0.027 | 0.130^a^ | 0.121^a^ | 0.478^a^ | 1.000 |

^a^The correlation is significant at a significance level of .01 (2-tailed).

^b^The correlation is significant at a significance level of .05 (2-tailed).

^c^Not applicable.

^d^The correlation is significant at a significance level of .1 (2-tailed).

**Table S4.** Results of collinearity diagnostics (cross-sectional study of physicians on Haodf.com, China, 2024, N=1,798).

| Variable | VIF | 1/VIF |
| --- | --- | --- |
| Self-disclosure breadth | 1.110 | 0.905 |
| Self-disclosure depth | 1.060 | 0.941 |
| DHL | 1.070 | 0.933 |
| Title | 1.090 | 0.919 |
| Popularity | 1.670 | 0.599 |
| Gift | 2.820 | 0.355 |

## Section 3: Robustness Check Results

Table S5 listed out continuous digital healthcare scores which were presented in the report published by [38].

**Table S5.** Digital healthcare scores and rankings of cities (cross-sectional study of physicians on Haodf.com, China, 2024, N=1,798).

| **Rank** | **City** | **Score** | **Rank** | **City** | **Score** | **Rank** | **City** | **Score** |
| --- | --- | --- | --- | --- | --- | --- | --- | --- |
| 1 | Beijing | 90.0 | 31 | Qingdao | 68.4 | 61 | Xingtai | 50.0 |
| 2 | Shanghai | 88.5 | 32 | Wuxi | 67.9 | 62 | Huainan | 49.2 |
| 3 | Guangzhou | 84.2 | 33 | Harbin | 67.1 | 63 | Jiangmen | 49.0 |
| 4 | Hangzhou | 83.6 | 34 | Lanzhou | 66.9 | 64 | Nantong | 48.7 |
| 5 | Zhengzhou | 81.8 | 35 | Quanzhou | 66.7 | 65 | Quzhou | 48.7 |
| 6 | Nanjing | 80.7 | 36 | Lianyungang | 65.9 | 66 | Linyi | 48.4 |
| 7 | Chengdu | 80.3 | 37 | Yinchuan | 65.9 | 67 | Lishui | 48.4 |
| 8 | Tianjin | 79.2 | 38 | Foshan | 64.7 | 68 | Jilin | 48.3 |
| 9 | Wuhan | 77.8 | 39 | Haikou | 64.4 | 69 | Jiaxing | 48.2 |
| 10 | Chongqing | 76.6 | 40 | Hohhot | 64.2 | 70 | Pingdingshan | 47.9 |
| 11 | Changsha | 76.2 | 41 | Taiyuan | 63.7 | 71 | Mianyang | 47.2 |
| 12 | Xiamen | 75.9 | 42 | Yantai | 63.4 | 72 | Handan | 47.2 |
| 13 | Shenzhen | 75.7 | 43 | Dongguan | 62.6 | 73 | Zhongshan | 46.7 |
| 14 | Ningbo | 75.2 | 44 | Wenzhou | 62.5 | 74 | Anqing | 46.7 |
| 15 | Shenyang | 74.7 | 45 | Xining | 60.2 | 75 | Liaocheng | 45.2 |
| 16 | Dalian | 74.2 | 46 | Changzhou | 59.4 | 76 | Jiaozuo | 45.2 |
| 17 | Changchun | 73.9 | 47 | Taizhou | 58.8 | 77 | Langfang | 44.8 |
| 18 | Guiyang | 73.1 | 48 | Zhenjiang | 58.7 | 78 | Yulin | 44.5 |
| 19 | Hefei | 72.9 | 49 | Yangzhou | 56.7 | 79 | Binzhou | 44.0 |
| 20 | Xi'an | 72.9 | 50 | Luoyang | 56.6 | 80 | Zigong | 43.9 |
| 21 | Nanchang | 71.8 | 51 | Zibo | 56.4 | 81 | Baoding | 43.4 |
| 22 | Nanning | 71.8 | 52 | Qinhuangdao | 55.8 | 82 | Chengde | 43.3 |
| 23 | Fuzhou | 70.5 | 53 | Taizhou | 53.0 | 83 | Deyang | 43.2 |
| 24 | Suzhou | 70.0 | 54 | Jiujiang | 51.7 | 84 | Yibin | 42.7 |
| 25 | Xuzhou | 69.9 | 55 | Liuzhou | 51.7 | 85 | Zhaoqing | 42.5 |
| 26 | Jinan | 69.9 | 56 | Suqian | 51.6 | 86 | Tai'an | 42.5 |
| 27 | Kunming | 69.8 | 57 | Fuyang | 51.4 | 87 | Dongying | 42.4 |
| 28 | Shijiazhuang | 69.8 | 58 | Weihai | 51.2 | 88 | Huizhou | 42.2 |
| 29 | Urumqi | 68.9 | 59 | Dezhou | 50.4 | 89 | Huai'an | 42.2 |
| 30 | Zhuhai | 68.4 | 60 | Chuzhou | 50.0 | 90 | Suining | 42.0 |
| 91 | Yancheng | 42.0 | 111 | Zunyi | 37.2 | 131 | Chizhou | 31.5 |
| 92 | Xuchang | 42.0 | 112 | Zhoushan | 37.2 | 132 | Wuhu | 31.2 |
| 93 | Huzhou | 42.0 | 113 | Hengyang | 37.2 | 133 | Cangzhou | 31.0 |
| 94 | Anshan | 42.0 | 114 | Baotou | 37.2 | 134 | Huaibei | 30.1 |
| 95 | Yueyang | 41.2 | 115 | Guilin | 36.9 | 135 | Kaifeng | 30.0 |
| 96 | Maoming | 40.7 | 116 | Tangshan | 34.2 | 136 | Ziyang | 29.7 |
| 97 | Changde | 40.5 | 117 | Zhuzhou | 34.2 | 137 | Heze | 29.5 |
| 98 | Lhasa | 39.9 | 118 | Yichang | 34.2 | 138 | Huangshan | 29.5 |
| 99 | Bengbu | 39.2 | 119 | Weifang | 34.2 | 139 | Guang'an | 29.2 |
| 100 | Tongling | 39.2 | 120 | Qiqihar | 34.2 | 140 | Jining | 29.2 |
| 101 | Shaoxing | 39.0 | 121 | Luzhou | 34.0 | 141 | Xuancheng | 29.2 |
| 102 | Neijiang | 39.0 | 122 | Ganzhou | 34.0 | 142 | Zhanjiang | 28.0 |
| 103 | Shantou | 39.0 | 123 | Zhangzhou | 34.0 | 143 | Suzhou | 27.1 |
| 104 | Nanchong | 39.0 | 124 | Leshan | 33.5 | 144 | Zhangjiakou | 26.2 |
| 105 | Nanyang | 39.0 | 125 | Bozhou | 32.2 | 145 | Meishan | 26.2 |
| 106 | Dazhou | 38.9 | 126 | Weinan | 32.1 | 146 | Lu'an | 26.2 |
| 107 | Xiangyang | 38.7 | 127 | Ma'anshan | 32.1 | 147 | Huai'an | 26.2 |
| 108 | Ordos | 38.2 | 128 | Jinhua | 32.1 | 148 | Baoji | 26.0 |
| 109 | Hengshui | 38.0 | 129 | Zhoukou | 31.9 | 149 | Mudanjiang | 24.7 |
| 110 | Xianyang | 38.0 | 130 | Daqing | 31.6 | 150 | Fushun | 23.0 |

**Table S6.** Robustness check 1 (R1) - Replace categorical DHL measurement with continuous digital healthcare scores (cross-sectional study of physicians on Haodf.com, China, 2024, N=1,798).

| Variables | Model 1  (95% CI) | Model 2  (95% CI) | Model 3  (95% CI) | Model 4  (95% CI) |
| --- | --- | --- | --- | --- |
| Self-disclosure breadth | 0.255^b^  (0.054-0.456) | 0.258^b^  (0.057-0.459) | __ | __ |
| Self-disclosure depth | __ | __ | 0.098^a^  (0.030-0.167) | 0.096^a^  (0.027-0.164) |
| DHL | __ | -0.003  (-0.007-0.001) | __ | -0.003  (-0.007-0.001) |
| Self-disclosure breadth $\text{×}$ DHL | __ | 0.020^a^  (0.006-0.034) | __ | __ |
| Self-disclosure depth $\text{×}$ DHL | __ | __ | __ | 0.006^b^  (0.001-0.010) |
| Title | 0.248^a^  (0.172-0.324) | 0.243^a^  (0.168-0.319) | 0.276^a^  (0.201-0.350) | 0.276^a^  (0.202-0.350) |
| Popularity | 1.720^a^  (1.503-1.936) | 1.757^a^  (1.538-1.976) | 1.678^a^  (1.460-1.896) | 1.705^a^  (1.484-1.926) |
| Gift | 0.001^a^  (0.001-0.001) | 0.001^a^  (0.001-0.001) | 0.001^a^  (0.001-0.001) | 0.001^a^  (0.001-0.001) |
| Constant | -1.825^a^  (-2.722-0.928) | -1.748^a^  (-2.876-1.062) | -1.748^a^  (-2.648-0.847) | -1.864^a^  (-2.776-0.951) |
| *R^2^* | 0.412 | 0.416 | 0.413 | 0.415 |
| *F* | 251.3 | 181.8 | 251.9 | 181.5 |

^a^The correlation is significant at a significance level of .01 (2-tailed).

^b^The correlation is significant at a significance level of .05 (2-tailed).

^c^Not applicable.

^d^The correlation is significant at a significance level of .1 (2-tailed).

**Table S7.** Robustness check 2 (R2) - Replace the dependent variable with Ln(Total visits-per-Popularity) (cross-sectional study of physicians on Haodf.com, China, 2024, N=1,798).

| Variables | Model 1  (95% CI) | Model 2  (95% CI) | Model 3  (95% CI) | Model 4  (95% CI) |
| --- | --- | --- | --- | --- |
| Self-disclosure breadth | 0.494^a^  (0.290-0.699) | 0.482^a^  (0.277-0.687) | __ | __ |
| Self-disclosure depth | __ | __ | 0.157^a^  (0.086-0.228) | 0.151^a^  (0.804-0.222) |
| DHL | __ | 0.021  (-0.035-0.769) | __ | 0.028  (-0.028-0.084) |
| Self-disclosure breadth $\times$ DHL | __ | 0.030^d^  (0.001-0.635) | __ | __ |
| Self-disclosure depth $\times$ DHL | __ | __ | __ | 0.071^d^  (0.001-0.142) |
| Title | 0.256^a^  (0.177-0.335) | 0.255^a^  (0.176-0.334) | 0.306^a^  (0.228-0.383) | 0.307^a^  (0.230-0.385) |
| Gift | 0.001^a^  (0.001-0.001) | 0.001^a^  (0.001-0.001) | 0.001^a^  (0.001-0.001) | 0.001^a^  (0.001-0.001) |
| Constant | 3.700^a^  (3.432-3.968) | 3.706^a^  (3.438-3.974) | 3.529^a^  (3.267-3.791) | 3.525^a^  (3.264-3.787) |
| *R^2^* | 0.324 | 0.325 | 0.323 | 0.324 |
| *F* | 214.9 | 144.0 | 213.6 | 143.4 |

^a^The correlation is significant at a significance level of .01 (2-tailed).

^b^The correlation is significant at a significance level of .05 (2-tailed).

^c^Not applicable.

^d^The correlation is significant at a significance level of .1 (2-tailed).

**Table S8.** Robustness check 3(R3) - Re-estimated all models using bootstrap resampling with 1,000 replications (cross-sectional study of physicians on Haodf.com, China, 2024, N=1,798).

| Variables | Model 1  (95% CI) | Model 2  (95% CI) | Model 3  (95% CI) | Model 4  (95% CI) |
| --- | --- | --- | --- | --- |
| Self-disclosure breadth | 0.255^b^  (0.050-0.460) | 0.249^b^  (0.049-0.448) | __ | __ |
| Self-disclosure depth | __ | __ | 0.098^a^  (0.029-0.167) | 0.092^a^  (0.026-0.158) |
| DHL | __ | -0.036  (-0.089-0.016) | __ | -0.031  (-0.085-0.022) |
| Self-disclosure breadth $\text{×}$ DHL | __ | 0.261^b^  (0.054-0.467) | __ | __ |
| Self-disclosure depth $\text{×}$ DHL | __ | __ | __ | 0.070^b^  (0.007-0.133) |
| Title | 0.248^a^  (0.176-0.320) | 0.242^a^  (0.168-0.315) | 0.276^a^  (0.201-0.350) | 0.275^a^  (0.200-0.350) |
| Popularity | 1.720^a^  (1.467-1.972) | 1.751^a^  (1.488-2.014) | 1.678^a^  (1.415-1.941) | 1.700^a^  (1.440-1.960) |
| Gift | 0.001^a^  (0.001-0.001) | 0.001^a^  (0.001-0.001) | 0.001^a^  (0.001-0.001) | 0.001^a^  (0.001-0.001) |
| Constant | -1.825^a^  (-2.865-0.785) | -1.938^a^  (-3.008-0.867) | -1.748^a^  (-2.827-0.668) | -1.838^a^  (-2.906-0.770) |
| *R^2^* | 0.412 | 0.415 | 0.413 | 0.415 |
| $\chi$^2^ | 920.56 | 917.50 | 936.66 | 922.84 |

^a^The correlation is significant at a significance level of .01 (2-tailed).

^b^The correlation is significant at a significance level of .05 (2-tailed).

^c^Not applicable.

^d^The correlation is significant at a significance level of .1 (2-tailed).

**Table S9.** Robustness check 4 - Restrict our analysis to non-chief physicians only (cross-sectional study of physicians on Haodf.com, China, 2024, N=1,798).

| Variables | Model 1  (95% CI) | Model 2  (95% CI) | Model 3  (95% CI) | Model 4  (95% CI) |
| --- | --- | --- | --- | --- |
| Self-disclosure breadth | 0.345^b^  (0.074-0.615) | 0.345^b^  (0.075-0.614) | __ | __ |
| Self-disclosure depth | __ | __ | 0.125^a^  (0.032-0.219) | 0.124^b^  (0.029-0.215) |
| DHL | __ | -0.083^b^  (-0.156-0.010) | __ | -0.092^b^  (-0.163-0.020) |
| Self-disclosure breadth $\text{×}$ DHL | __ | 0.259^b^  (0.001-0.518) | __ | __ |
| Self-disclosure depth $\text{×}$ DHL | __ | __ | __ | 0.086^b^  (0.001-0.173) |
| Title | 0.204^a^  (0.051-0.357) | 0.187^b^  (0.034-0.340) | 0.255^a^  (0.103-0.406) | 0.236^a^  (0.085-0.387) |
| Popularity | 1.919^a^  (1.616-2.221) | 2.013^a^  (1.704-2.322) | 1.863^a^  (1.558-2.170) | 1.952^a^  (1.641-2.266) |
| Gift | 0.001^a^  (0.001-0.002) | 0.001^a^  (0.001-0.002) | 0.002^a^  (0.001-0.002) | 0.002^a^  (0.001-0.002) |
| Constant | -2.538^a^  (-3.788-1.288) | -2.877^a^  (-4.145-1.608) | -2.468^a^  (-3.725-1.217) | -2.788^a^  (-4.062-1.514) |
| *R^2^* | 0.411 | 0.418 | 0.411 | 0.418 |
| *F* | 128.3 | 94.12 | 128.6 | 94.20 |

^a^The correlation is significant at a significance level of .01 (2-tailed).

^b^The correlation is significant at a significance level of .05 (2-tailed).

^c^Not applicable.

^d^The correlation is significant at a significance level of .1 (2-tailed).

**Table S10.** Robustness check 5 - Re-estimated all models after winsorizing the top and bottom 10% of each variable (cross-sectional study of physicians on Haodf.com, China, 2024, N=1,798).

| Variables | Model 1  (95% CI) | Model 2  (95% CI) | Model 3  (95% CI) | Model 4  (95% CI) |
| --- | --- | --- | --- | --- |
| Self-disclosure breadth | 0.250^b^  (0.030-0.470) | 0.248^b^  (0.028-0.468) | __ | __ |
| Self-disclosure depth | __ | __ | 0.106^a^  (0.032-0.180) | 0.101^a^  (0.261-0.175) |
| DHL | __ | -0.037  (-0.091-0.017) | __ | -0.032  (-0.085-0.022) |
| Self-disclosure breadth $\text{×}$ DHL | __ | 0.285^b^  (0.066-0.504) | __ | __ |
| Self-disclosure depth $\text{×}$ DHL | __ | __ | __ | 0.076^b^  (0.003-0.149) |
| Title | 0.250^a^  (0.174-0.326) | 0.243^a^  (0.167-0.319) | 0.276^a^  (0.201-0.350) | 0.275^a^  (0.201-0.349) |
| Popularity | 1.719^a^  (1.502-1.935) | 1.750^a^  (1.531-1.970) | 1.679^a^  (1.461-1.900) | 1.701^a^  (1.480-1.942) |
| Gift | 0.001^a^  (0.001-0.001) | 0.001^a^  (0.001-0.001) | 0.001^a^  (0.001-0.001) | 0.001^a^  (0.001-0.001) |
| Constant | -1.827^a^  (-2.725-0.930) | -1.940^a^  (-2.847-1.033) | -1.753^a^  (-2.653-0.853) | -1.842^a^  (-2.753-0.931) |
| *R^2^* | 0.412 | 0.415 | 0.413 | 0.415 |
| *F* | 250.9 | 181.1 | 251.9 | 181.1 |

^a^The correlation is significant at a significance level of .01 (2-tailed).

^b^The correlation is significant at a significance level of .05 (2-tailed).

^c^Not applicable.

^d^The correlation is significant at a significance level of .1 (2-tailed).

## References

1. Faghihnasiri K, Alves HMB, Soares AM. Value Co-Creation Activities Role in Patient Well-Being in Online Healthcare Communities. In: Soares AM, Casais B, editors. Uniting Marketing Efforts for the Common Good—A Challenge for the Fourth Sector Cham: Springer International Publishing; 2023. p. 149–175. doi: 10.1007/978-3-031-29020-6_8

2. Min J, Chen Y, Wang L, He T, Tang S. Diabetes self-management in online health communities: an information exchange perspective. BMC Med Inform Decis Mak 2021 Jun 28;21(1):201. doi: 10.1186/s12911-021-01561-3

3. Jiang H, Mi Z, Xu W. Online Medical Consultation Service–Oriented Recommendations: Systematic Review. Journal of Medical Internet Research 2024 Jul 30;26(1):e46073. doi: 10.2196/46073

4. Li H. Asymmetric Information in the Field of Healthcare. Advances in Economics, Management and Political Sciences 2024 Jul 23;100:28–34. doi: 10.54254/2754-1169/100/20241089

5. Zhang L, Zhan J, Wan VKW, Wang Y. Designing and Evaluating Online Health Consultation Interfaces: A Perspective of Physician-Patient Power Asymmetry. IEEE Access 2024;12:124111–124127. doi: 10.1109/ACCESS.2024.3454213

6. Zhang M, Sun Y, Zhao X, Wang L, Xiong J. The Impact of Narrative Reviews on Patient E-doctor Choice in Online Health Communities. INQUIRY SAGE Publications Inc; 2023 Jan 1;60:00469580231183695. doi: 10.1177/00469580231183695

7. Simons G, Baldwin DS. A critical review of the definition of ‘wellbeing’ for doctors and their patients in a post Covid-19 era. Int J Soc Psychiatry SAGE Publications Ltd; 2021 Dec 1;67(8):984–991. doi: 10.1177/00207640211032259

8. Chen Y, Lee S. User-Generated Physician Ratings and Their Effects on Patients’ Physician Choices: Evidence from Yelp. Journal of Marketing SAGE Publications Inc; 2024 Jan;88(1):77–96. doi: 10.1177/00222429221146511

9. Song M, Elson J, Bastola D. Digital Age Transformation in Patient-Physician Communication: 25-Year Narrative Review (1999-2023). Journal of Medical Internet Research 2025 Jan 16;27(1):e60512. doi: 10.2196/60512

10. Lu W, Wu H. How Online Reviews and Services Affect Physician Outpatient Visits: Content Analysis of Evidence From Two Online Health Care Communities. JMIR Med Inform 2019 Dec 2;7(4):e16185. doi: 10.2196/16185

11. Sze KP, Fong QW, De Roza JG, Lee ES, Tan SY. Exploring Physicians’ Perceptions of Digital Health’s Impact on the Patient-Physician Relationship in the Primary Health Care Setting: Qualitative Descriptive Study. J Med Internet Res 2024 Oct 15;26:e53705. doi: 10.2196/53705

12. Lu X. The Effects of Patient Health Information Seeking in Online Health Communities on Patient Compliance in China: Social Perspective. J Med Internet Res 2023 Jan 9;25:e38848. PMID:36622741

13. Huang Z, Duan C, Yang Y, Khanal R. Online selection of a physician by patients: the impression formation perspective. BMC Medical Informatics and Decision Making 2022 Jul 25;22(1):193. doi: 10.1186/s12911-022-01936-0

14. Ma L, Ding X, Zhang X, Zhang G. Mobile Users’ Self-Disclosure Behaviour on WeChat: Application of Social Cognitive Theory. Mobile Information Systems 2020 Aug 1;2020:1–13. doi: 10.1155/2020/8903247

15. Yang X, Huang Y, Li B. Attachment anxiety and cyberbullying victimization in college students: the mediating role of social media self-disclosure and the moderating role of gender. Front Psychol 2023 Nov 14;14:1274517. doi: 10.3389/fpsyg.2023.1274517

16. Montgomery T, Berns JS, Braddock CH III. Transparency as a Trust-Building Practice in Physician Relationships With Patients. JAMA 2020 Dec 15;324(23):2365–2366. doi: 10.1001/jama.2020.18368

17. Papneja H, Yadav N. Self-disclosure to conversational AI: a literature review, emergent framework, and directions for future research. Pers Ubiquit Comput 2025 Apr 1;29(2):119–151. doi: 10.1007/s00779-024-01823-7

18. Jo E, Jeong Y, Park S, Epstein DA, Kim Y-H. Understanding the Impact of Long-Term Memory on Self-Disclosure with Large Language Model-Driven Chatbots for Public Health Intervention. Proceedings of the 2024 CHI Conference on Human Factors in Computing Systems New York, NY, USA: Association for Computing Machinery; 2024. p. 1–21. doi: 10.1145/3613904.3642420

19. Li C, Wang C, Chau PYK. Revealing the black box: Understanding how prior self-disclosure affects privacy concern in the on-demand services. International Journal of Information Management 2022 Dec;67:102547. doi: 10.1016/j.ijinfomgt.2022.102547

20. Ferrell D, Campos-Castillo C. Factors Affecting Physicians’ Credibility on Twitter When Sharing Health Information: Online Experimental Study. JMIR Infodemiology 2022 Jun 13;2(1):e34525. doi: 10.2196/34525

21. Al Mufarreh R. How much, what and how: three-dimensional discourse analysis of Saudi women and men’s self-disclosure. SJLS 2023 Nov 21;3(4):200–219. doi: 10.1108/SJLS-04-2023-0016

22. Obermaier M, Schmuck D. Youths as targets: factors of online hate speech victimization among adolescents and young adults. Vitak J, editor. Journal of Computer-Mediated Communication 2022 Jul 14;27(4):zmac012. doi: 10.1093/jcmc/zmac012

23. Zulman DM, Haverfield MC, Shaw JG, Brown-Johnson CG, Schwartz R, Tierney AA, Zionts DL, Safaeinili N, Fischer M, Thadaney Israni S, Asch SM, Verghese A. Practices to Foster Physician Presence and Connection With Patients in the Clinical Encounter. JAMA 2020 Jan 7;323(1):70–81. doi: 10.1001/jama.2019.19003

24. Liu J, He J, He S, Li C, Yu C, Li Q. Patients’ Self-Disclosure Positively Influences the Establishment of Patients’ Trust in Physicians: An Empirical Study of Computer-Mediated Communication in an Online Health Community. Front Public Health Frontiers; 2022 Jan 25;10. doi: 10.3389/fpubh.2022.823692

25. Bismire H, Nunn S, Malpas CB, Bilszta JL. Doctor Who? Honorific titles and their influence on patients’ perceptions of healthcare professionals. J R Soc Med SAGE Publications; 2022 Mar 1;115(3):91–94. doi: 10.1177/01410768221080775

26. Catapan S de C, Taylor ML, Scuffham P, Smith AC, Kelly JT. Improving consumer trust in digital health: A mixed methods study involving people living with chronic kidney disease. Digit Health 2025 Jan 9;11:20552076241312440. PMID:39801583

27. Oh YJ, Ryu JY, Han J, Park S, Lim JI, Youk S, Park HS. Trust in AI Doctors: How Credibility and Message Richness in AI-Based Providers Influence Patient Adherence. Advances in Human-Computer Interaction 2025;2025(1):5934127. doi: 10.1155/ahci/5934127

28. Phi NTT, Montori VM, Kunneman M, Ravaud P, Tran V-T. Cumulative Burden of Digital Health Technologies for Patients With Multimorbidity: A Systematic Review. JAMA Netw Open 2025 Apr 1;8(4):e257288. PMID:40279126

29. Jarva E, Oikarinen A, Andersson J, Tuomikoski A-M, Kääriäinen M, Meriläinen M, Mikkonen K. Healthcare professionals’ perceptions of digital health competence: A qualitative descriptive study. Nursing Open 2022;9(2):1379–1393. doi: 10.1002/nop2.1184

30. Zhang H, Xiao Y, Zhao X, Tian Z, Zhang S, Dong D. Physicians’ knowledge on specific rare diseases and its associated factors: a national cross-sectional study from China. Orphanet J Rare Dis 2022 Mar 5;17:120. PMID:35248110

31. Catapan S de C, Sazon H, Zheng S, Gallegos-Rejas V, Mendis R, Santiago PHR, Kelly JT. A systematic review of consumers’ and healthcare professionals’ trust in digital healthcare. NPJ Digit Med 2025 Feb 21;8:115. PMID:39984678

32. Dave P. How Digital Health is Revolutionizing Healthcare and Contributing to Positive Health Outcomes. J Drug Delivery Ther 2024 Jun 15;14(6):287–293. doi: 10.22270/jddt.v14i6.6640

33. Miao M, Morrow R, Salomon A, Mcculloch B, Evain J-C, Wright MR, Murphy MT, Welsh M, Williams L, Power E, Rietdijk R, Debono D, Brunner M, Togher L. Digital Health Implementation Strategies Coproduced With Adults With Acquired Brain Injury, Their Close Others, and Clinicians: Mixed Methods Study With Collaborative Autoethnography and Network Analysis. J Med Internet Res 2023 Sep 19;25:e46396. PMID:37725413

34. Riemann JF, Teufel A. Digital Communication Strategies in Visceral Medicine. Visc Med 2021 Dec;37(6):455–457. PMID:35087895

35. Nazi KM. The Future of Personal Health Records and Patient Portals. Medical Research Archives 2021 Dec 27;9(12). doi: 10.18103/mra.v9i12.2641

36. Naumann A, Tappe U, Teufel A. Digital Technology in Visceral Medicine: An Overview in Outpatient Care in Germany. Visc Med 2021 Dec;37(6):465–470. PMID:35087896

37. DiPiro JT, Hoffman JM, Tichy E, Shea S, Sanborn M, Hung A, Fox ER, Watanabe JH, Torrise V, Abourjaily P, Cunningham FE, Schweitzer P, Nelson SD, Stump LS, Castro H, Nesbit TW, Scott CM. ASHP and ASHP Foundation Pharmacy Forecast 2025: Strategic Planning Guidance for Pharmacy Departments in Hospitals and Health Systems. Am J Health Syst Pharm 2025 Jan 6;82(2):17–47. PMID:39657700

38. China Urban Digital Economy Index (Medical Chapter) (2021). Available from: https://roadshow.h3c.com/zl/pdf/ylbps.pdf [accessed Nov 15, 2025]
